# Human genetics and malaria resistance

**DOI:** 10.1007/s00439-020-02142-6

**Published:** 2020-03-04

**Authors:** Silvia N. Kariuki, Thomas N. Williams

**Affiliations:** 1 Department of Epidemiology, KEMRI-Wellcome Trust Research Programme, Kilifi, Kenya; 2 Department of Medicine, Imperial College of Science and Technology, London, UK

## Abstract

Malaria has been the pre-eminent cause of early mortality in many parts of the world throughout much of the last five thousand years and, as a result, it is the strongest force for selective pressure on the human genome yet described. Around one third of the variability in the risk of severe and complicated malaria is now explained by additive host genetic effects. Many individual variants have been identified that are associated with malaria protection, but the most important all relate to the structure or function of red blood cells. They include the classical polymorphisms that cause sickle cell trait, α-thalassaemia, G6PD deficiency, and the major red cell blood group variants. More recently however, with improving technology and experimental design, others have been identified that include the Dantu blood group variant, polymorphisms in the red cell membrane protein *ATP2B4*, and several variants related to the immune response. Characterising how these genes confer their effects could eventually inform novel therapeutic approaches to combat malaria. Nevertheless, all together, only a small proportion of the heritable component of malaria resistance can be explained by the variants described so far, underscoring its complex genetic architecture and the need for continued research.

## Introduction

Malaria has been the biggest cause of childhood mortality globally for much of the last 5000 years. Although now coming under some degree of control, mortality remains high in many countries and worldwide there were an estimated 405,000 deaths from malaria in 2018 alone, with more than 90% of these deaths occurring in sub-Saharan Africa (WHO 2019). Historically, this pressure has resulted in the selection of a wide range of genetic variants that confer protection against malaria-specific death. This review aims to outline some of the more important protective genetic variants that have been identified so far, as summarized in the Table 1. Understanding how these variants confer their protective effects has the potential to inform novel preventative and treatment approaches.

**Table 1:** A summary of the malaria protective gene variants that are reviewed, with key references

Gene	Encoded protein	Variant	Mechanistic hypotheses	References
*HBB*	β-globin	Heterozygous carriers of sickle haemoglobin (HbAS)	Increased clearance of sickled infected RBCs by the spleen Acquired host immunity and increased phagocytosis of ring-parasitised variant RBCs Reduced cytoadherence and rosetting Impaired trafficking of parasite proteins to RBC surface Inhibition of parasite growth due to oxygen-dependent polymerization of sickle haemoglobin	Mackey and Vivarelli (1954), Miller, Neel, and Livingstone (1956), Luzzatto, Nwachuku-Jarrett, and Reddy (1970) Williams et al. (2005), Ayi et al. (2004) Carlson et al. (1994), Cholera et al. (2008), Opi et al. (2014) Cyrklaff et al. (2011), McAuley et al. (2010), Komba et al. (2009), Makani et al. (2010), Archer et al. (2018)
*HBB*	β-globin	Heterozygous β-thalassaemia (absent or reduced β-globin)	Enhanced antibody binding and subsequent clearance of infected variant RBCs Increased phagocytosis of ring-parasitised variant RBCs	Luzzi, Merry, Newbold, Marsh, Pasvol, et al. (1991a, b), Ayi et al. (2004)
*HBA*	α-globin	α-thalassaemia (deletion or inactivation of one or more of the normal 4 α-globin genes)	Increased phagocytosis of infected variant RBCs by monocytes Enhanced antibody binding and subsequent clearance of infected variant RBCs	Yuthavong et al. (1988), Yuthavong, Bunyaratvej, and Kamchonwongpaisan (1990) Luzzi, Merry, Newbold, Marsh, and Pasvol (1991a, b)
*G6PD*	Glucose-6-phosphate dehydrogenase (G6PD)	Female heterozygotes for G6PD deficiency (G6PDd)	Increased phagocytosis of ring-parasitised variant RBCs due to enhanced oxidative stress	Cappadoro et al. (1998), Ayi et al. (2004)
*CR1*	Complement Receptor One	Swain-Langley 2 (Sl2) polymorphism	Reduced binding of Sl2 RBCs to the parasite rosetting ligand PfEMP1	Rowe et al. (1997), Opi et al. (2018)
*FY*	Duffy antigen receptor for chemokines (DARC)	FY*ES allele	Inhibition of *P. vivax* invasion of Duffy negative RBCs through impaired junction formation	Miller et al. (1975), Miller et al. (1976), Miller et al. (1979), Grimberg et al. (2007)
*ABO*	Glycosyltransferase enzyme	*ABO* single nucleotide deletion (rs8176719)—Blood group O	Reduced *P. falciparum* rosetting	Rowe et al. (2007), Rowe et al. (1995), Udomsangpetch et al. (1993), MalariaGEN (2014), Ndila et al. (2018)
*ATP2B4*	PMCA4 calcium transporter	*ATP2B4* single nucleotide polymorphisms (rs4951074 and rs1541255)	Altered binding of transcription factors to *ATP2B4* enhancer elements, leading to decreased gene expression, and subsequent dysregulated intracellular calcium homeostasis	Zambo et al. (2017), Gazarini et al. (2003), Tiffert et al. (2005), Lessard et al. (2017), MalariaGEN (2014), Ndila et al. (2018)
*GYP*	Glycophorins	Duplicate *GYPB-A* hybrid genes encoding the Dantu blood group	Inhibition of *P. falciparum* invasion due to increased membrane tension of Dantu variant RBCs	Band et al. (2015), Leffler et al. (2017), Ndila et al. (2018), Algady et al. (2018), Kariuki et al. (2018)
*IL23R, IL12-RB2*	Interleukin 23 and Interleukin 12 receptor complex	*IL23R*-*IL12RB2* single nucleotide polymorphism clusters	Immunoregulatory roles in protective immunity in malaria infections	Ravenhall et al. (2018), Luty et al. (2000), Malaguarnera et al. (2002), Ong'echa et al. (2008), Zhang et al. (2010), Munde et al. (2017)

**Fig. 1:**
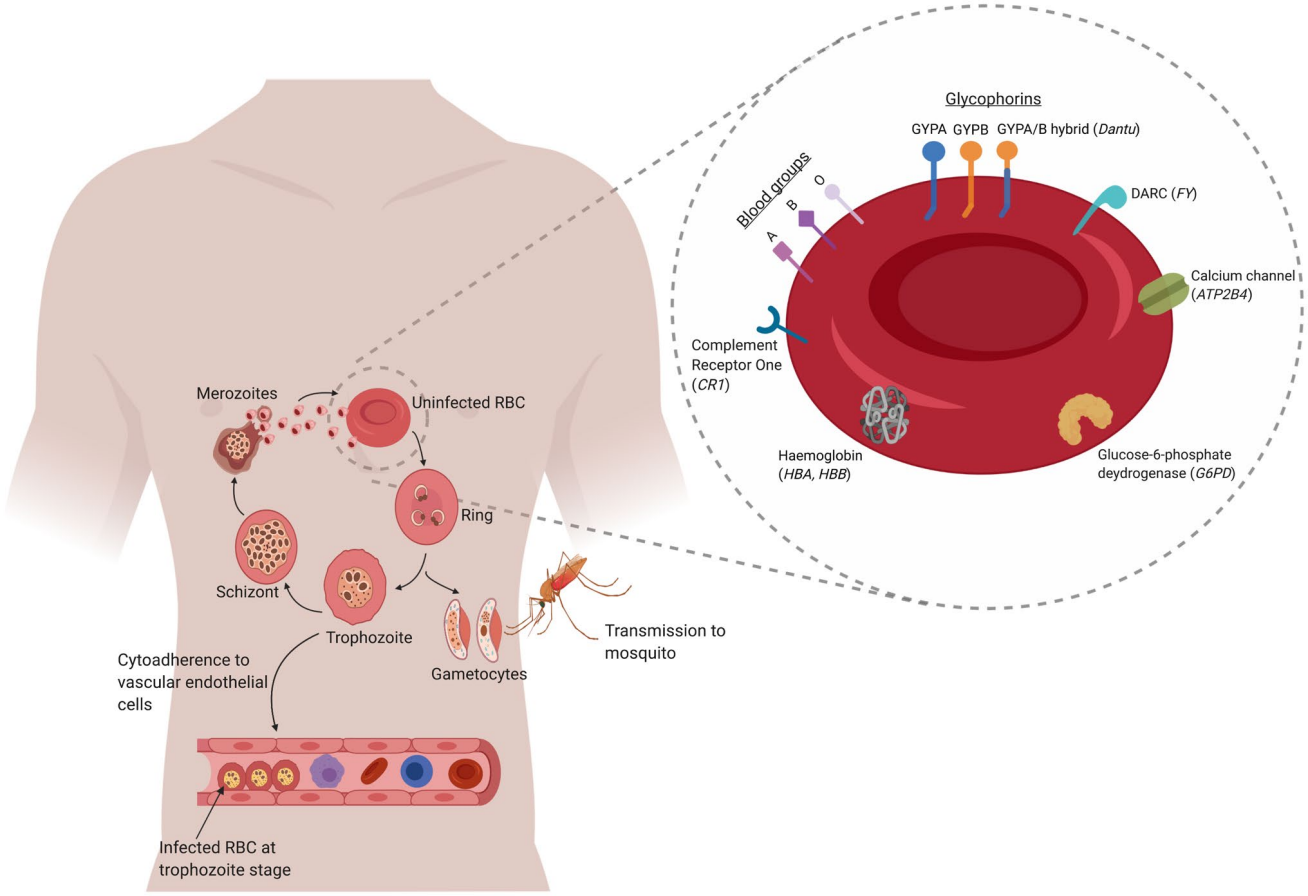
The blood-stage of the *P. falciparum* life cycle in the human host. Inset: illustration of the malaria-protective variants that have important roles in the red blood cell (RBC). Image made using ©BioRender (https://biorender.com)

Malaria can be caused by five different species of protozoan *Plasmodium* parasites: *P. falciparum*, *P. vivax*, *P. malariae*, *P. ovale* and *P. knowlesi*. *P. falciparum* and *P. vivax* are the most prevalent and result in the majority of deaths (Escalante et al. 1995; Singh et al. 2004; Martinsen et al. 2008; WHO 2019). The propagation of all malaria parasites involves both a sexual cycle in mosquitoes and an asexual cycle in humans, in which parasites first infect the liver before becoming established in red blood cells (RBCs). The clinically symptomatic blood-stage involves sequential rounds of parasite multiplication that incorporate invasion of merozoites into RBCs followed by their maturation into trophozoites then schizonts, which ultimately rupture from the RBC to release fresh merozoites which rapidly infect new RBCs (Fig. 1). The asexual cycle gives rise to an exponential multiplication of parasites in the blood and to the pathogenic features of malaria that are observed in the human host (Schellenberg et al. 1994; Rogier et al. 1999; Snow et al. 2005). The parasite is able to evade the host immune response by sequestering in the deep capillaries (Rowe et al. 1995, 2009; Williams et al. 2002; Butthep et al. 2006). There are multiple points in the parasite lifecycle that have impacted host genetic variation, but the majority of the malaria-protective variants described so far have various important impacts on the structure and function of the RBC (Fig. 1).

## The selective force of malaria on the human genome

The “malaria hypothesis” was first proposed by JBS Haldane more than seventy years ago (Haldane 1949). He speculated that the reason why thalassaemia was common in the Mediterranean region was because it conferred a survival advantage against malaria. At around the same time, Allison (Allison 1954) and others were speculating that malaria selection might also have explained the high frequency of haemoglobin S (HbS) in malaria endemic areas. Both the thalassaemias and HbS are disorders of haemoglobin that are caused by various mutations in the α- and β-globin genes (*HBA* and *HBB*). It took many years for the veracity of these hypotheses to be established beyond reasonable doubt, but it is now clear that the HbS variant confers the strongest protective effect against severe malaria that has yet been described, with an effect size of  > 80% in heterozygous carriers (HbAS*;* sickle cell trait), while α-thalassaemia confers a protective effect of approximately 40% in homozygotes (MalariaGEN 2014; Ndila et al. 2018). Other protective red blood cell (RBC) polymorphisms have also been shown to occur at their highest frequencies in malaria endemic populations, including glucose-6-phosphatase (G6PD) deficiency, the O blood group, and variants of the gene for complement receptor 1 (*CR1*) (Kwiatkowski 2005; Williams 2016; Rowe et al. 1997; Opi et al. 2018).

One of the first and most conclusive examples of malaria effecting a strong selective pressure on the human genome is the Duffy antigen receptor for chemokines (DARC), which is expressed on red blood cell membranes and has roles as both a chemokine receptor (Horuk et al. 1993; Pogo and Chaudhuri 1995; Hadley and Peiper 1997) and an invasion receptor for *Plasmodium vivax* merozoites (Miller et al. 1975, 1976; Wertheimer and Barnwell 1989; Adams et al. 1990; Chitnis and Miller 1994; Grimberg et al. 2007). DARC is encoded by the Duffy blood group *FY* gene, that occurs in the form of three common alleles with starkly differing global allele frequency distributions: FY*A, FY*B and the “erythrocyte silent” FY*ES. The FY*ES allele, which results in the absence of the Duffy antigen, is found at frequencies nearing fixation in sub-Saharan Africa but is virtually absent in non-African populations (Hamblin and Di Rienzo 2000; Howes et al. 2011). The allele frequency difference of the FY*ES allele across populations is the largest difference observed in the human genome to date (Cavalli-Sforza, Menozzi, and Piazza 1994), a strong indicator of positive natural selection (Hamblin and Di Rienzo 2000). These and similar data suggest that malaria has been responsible for exerting the strongest selective pressure on the human genome that has so far been described (Flint et al. 1998; Tishkoff and Williams 2002).

## Heritability of malaria

The extent to which variation in the incidence of malaria is attributed to host genetic factors has been investigated in a number of studies. Using pedigree-based variance component analysis, studies conducted in Sri Lanka (Mackinnon et al. 2000), Kenya (Mackinnon et al. 2005), Senegal (Sakuntabhai et al. 2008) and Thailand (Phimpraphi et al. 2008) have established that additive genetic factors explain approximately one quarter of the total variation in the incidence of uncomplicated *P. falciparum* malaria and more than one third of the variation in severe and complicated disease. However, only 2% of this variance appears to be explained by HbS and α-thalassaemia together, two of the most important polymorphisms discovered so far in terms of their frequencies and effect sizes (Mackinnon et al. 2005). This indicates that the genetic architecture of malaria susceptibility is much more complex than is currently understood and that “missing heritability” might yet be explained by polygenetic or epigenetic effects, or by gene–gene and gene-environment interactions (Manolio et al. 2009).

## Protective loci identified by linkage and genomic epidemiology

Malaria associated genes have been identified through numerous approaches including family-based studies that have linked broad chromosomal regions to the risk of malaria parasite carriage (Garcia et al. 1998; Rihet et al. 1998; Flori et al. 2003a, b; Timmann et al. 2007). Such early studies were, however, limited in their ability to fine-map the specific gene variants underlying the broad chromosomal linkage signals. More recently, genomic epidemiology approaches such as case–control and cohort studies have focused on characterising the allele frequency distributions, effect sizes and directions of effect of various candidates including HbS, α-thalassaemia, G6PD deficiency, and the ABO blood group locus (Allison 1954; Bienzle et al. 1972; Ruwende et al. 1995; Wambua et al. 2006; MalariaGEN 2014). While many have now been shown to be associated with significant effects, recent studies suggest that these known candidate genes only explain a small fraction of the heritability of malaria and that there could be many other genetic variants that are unaccounted for by the single-gene study approach (Mackinnon et al. 2005; Verra, Mangano, and Modiano 2009; Damena et al. 2019).

## Novel resistance loci identified by genome-wide association studies

Recent genome-wide association studies (GWAS) in malaria endemic populations have confirmed many of the classical malaria associated genes (MalariaGEN 2014, 2019) and enabled the identification of additional novel associations (Jallow et al. 2009; Timmann et al. 2012; Band et al. 2015; Ravenhall et al. 2018; MalariaGEN 2019). There have been various challenges with performing GWAS in African populations (Teo et al. 2010; Damena et al. 2019). Africans have high levels of genomic diversity due to their long ancestral history and, compared to non-African populations, their genomes are characterized by shorter linkage disequilibrium blocks between loci (Tishkoff and Williams 2002; Conrad et al. 2006; Campbell and Tishkoff 2008; Jakobsson et al. 2008; Tishkoff et al. 2009). The genotyping platforms that were used in early GWAS studies therefore had low tagging efficiency in these populations and resulted in relatively weak associations, even at some of the best known malaria associated loci such as HbS (Jallow et al. 2009). Furthermore, few analyses have considered interactions between genes and even fewer have incorporated genomic data from the parasites or vectors that might be relevant to patient outcomes (Damena et al. 2019). While such approaches are now becoming increasingly feasible from a computational perspective, they are currently limited by the availability of such rich phenotypic data—an aspiration for future studies. In the meantime, the performance of human-only GWAS studies have been substantially improved by the imputation of missing variants through the inclusion in reference panels of more diverse African populations (Band et al. 2013; Gurdasani et al. 2015, 2019; MalariaGEN 2019), the use of customised representative genotyping platforms that better capture the genomic diversity of African populations (Gurdasani et al. 2015, 2019; Johnston et al. 2017) and the additional deep sequencing of target loci (Jallow et al. 2009; Band et al. 2015; Leffler et al. 2017). In a recent study, the inclusion of a denser reference panel in combination with sequence data from Phase 3 of the 1000 Genomes Project (Auton et al. 2015) significantly improved the quality of variant calling in one severe malaria GWAS, including the identification of copy number variants that had not been detected in an earlier analysis (Leffler et al. 2017). Such improvements have recently led to the identification of new associations, including variants in *ATP2B4* which encodes the major calcium transporter in RBCs, PMCA4, and confers a 40% protective effect (Timmann et al. 2012; Band et al. 2015; MalariaGEN 2019). The *ATP2B4* variants lead to reduced expression of the PMCA4 protein, possibly due to altered binding of transcription factors that regulate PMCA4 expression (Zambo et al. 2017; MalariaGEN 2019). This reduced PMCA4 expression could lead to alterations in intracellular calcium homeostasis and affect the development of parasites during their intra-erythrocytic lifecycle (Gazarini et al. 2003; Tiffert et al. 2005). More functional studies are required to elucidate the exact protective mechanisms.

A second recently described novel malaria resistance gene involves a complex structural rearrangement in the glycophorin gene cluster that results in the gain of two *GYPB-A* hybrid genes to encode the Dantu blood group antigen (Leffler et al. 2017). Glycophorins are sialoglycoproteins that are abundantly expressed on the surface of RBCs and that bear the antigenic determinants of the MNS blood groups (Blumenfeld and Huang 1995, 1997). This locus provides an exciting potential therapeutic target for *P. falciparum* therapies because, akin to the story of *P. vivax* malaria, the glycophorins have been shown to act as invasion ligands for the Duffy-Binding-Like (DBL) domains of a range of *P. falciparum* merozoite proteins (Sim et al. 1994; Tolia et al. 2005; Mayer et al. 2001, 2002, 2004, 2006, 2009). In homozygotes, Dantu confers a strongly protective effect size of 74% against all forms of severe falciparum malaria (Band et al. 2015, Leffler et al. 2017, Ndila et al. 2018, MalariaGEN 2019). Curiously, this polymorphism is found at highest frequencies in East Africa, specifically in the coastal region of Kilifi, and is rare or absent in other malaria endemic regions. One possible explanation is that positive selection for the Dantu polymorphism by malaria might historically have been balanced by increased mortality from other diseases. Interestingly, features of ancient balancing selection are seen at this locus (Leffler et al. 2013; Band et al. 2015), underscoring the fact that malaria could be one of a number of opposing evolutionary driving forces acting on the glycophorin region, a question that is currently being addressed in ongoing studies.

In a recent GWAS conducted in north-east Tanzania, novel variants were identified in the immune genes *IL-23R* and *IL-12RB2* which were specifically found to be associated with protection against severe malaria anaemia (Ravenhall et al. 2018). These genes encode vital pro-inflammatory cytokine receptors which have important immunoregulatory roles in protective immunity against malaria infections (Luty et al. 2000; Malaguarnera et al. 2002; Ong'echa et al. 2008; Zhang et al. 2010; Munde et al. 2017). In the same cohort, signals of recent positive selection were also found at several loci within the MHC region, immune-related genes that could potentially inform malaria vaccine development.

## Functional validation of malaria-protective genes

Beyond identifying malaria-protective gene variants, investigations into the mechanisms through which these variants confer their protective effects are critical to informing novel approaches to intervention. Functional studies have led to the elucidation of key steps in the molecular processes involved in parasite invasion of host RBCs, with the seminal example of the *FY* gene that encodes DARC (formerly known as the Duffy blood group system of antigens). This discovery led to further functional studies that identified the *P. vivax* Duffy-binding protein (*Pv*DBP) that is crucial for RBC invasion (Miller et al. 1979; Haynes et al. 1988; Wertheimer and Barnwell 1989; Chitnis and Miller 1994), which is now undergoing clinical trials as a vaccine candidate (Chitnis and Sharma 2008; Mueller, Shakri, and Chitnis 2015). Cases of *P. vivax* infection in FY*ES individuals have more recently been reported (Ryan et al. 2006; Menard et al. 2010; Ngassa Mbenda and Das 2014; Lo et al. 2015; Abdelraheem et al. 2016; Niangaly et al. 2017). Functional work leading to the discovery of transferrin receptor 1 (TfR1) as an important alternative receptor for *P. vivax* recognition and invasion of RBCs could explain these cases (Gruszczyk et al. 2018). TfR1 is a receptor for the *P. vivax* reticulocyte binding protein 2b (PvRBP2b) and as such it offers a potential alternative vaccine target.

In the case of the most strongly protective variant against *P. falciparum*, HbAS, several mechanisms of protection have been proposed, including sickling of the infected RBCs (Mackey and Vivarelli 1954; Miller, Neel, and Livingstone 1956), leading to increased clearance by the spleen (Luzzatto, Nwachuku-Jarrett, and Reddy 1970), impaired haemoglobin digestion (Pasvol, Weatherall, and Wilson 1978; Pasvol 1980; Friedman 1978), and acquired host immunity (Williams et al. 2005). More recently, Cyrklaff et al. showed that the actin cytoskeleton network that directs RBC trafficking of parasite encoded proteins, such as the *P. falciparum* erythrocyte membrane protein-1 (PfEMP1), was impaired in HbAS RBCs (Cyrklaff et al. 2011). Impaired trafficking of parasite proteins to the surface of the RBC could explain the observation that cytoadherence of parasitised RBCs to the vascular endothelium, and binding of parasitised RBCs to uninfected RBCs to form rosettes, are both significantly reduced in HbAS RBCs (Carlson et al. 1994; Cholera et al. 2008; Opi et al. 2014). The latter observation is akin to that postulated as the protective mechanism for blood group O (Rowe et al. 1995, 2007; Udomsangpetch et al. 1993). Furthermore, impaired parasite growth and development in HbAS RBCs has also been reported (McAuley et al. 2010; Komba et al. 2009; Makani et al. 2010), with one recent study demonstrating that oxygen-dependent polymerization of HbS is responsible for *P. falciparum* growth inhibition (Archer et al. 2018). Finally, immune-mediated protective mechanisms have also been postulated for HbAS, as well as α- and β-thalassaemias, and G6PD deficiency. These include enhanced antibody binding and phagocytosis of infected variant RBCs, possibly due to oxidative damage of the RBC membrane (Yuthavong et al. 1988,1990; Luzzi et al. 1991a, b; Ayi et al. 2004; Cappadoro et al. 1998).

Technological advances have further aided the functional validation efforts for newly identified malaria-protective variants. Lessard et al. investigated the *ATP2B4* locus in detail using transcriptomics, epigenomics, and gene-editing, and found that the *ATP2B4* GWAS SNPs mapped to enhancer elements that regulated *ATP2B4* gene expression and subsequent intracellular calcium homeostasis (Lessard et al. 2017). Functional annotation of the malaria-protective *ATP2B4* SNPs in the recent GWAS carried out by the Malaria Genomic Epidemiology Network also found that these SNPs regulate *ATP2B4* gene expression by disrupting the promoter upstream of the gene’s transcription start site (MalariaGEN 2019). Similarly, since the protective association of Dantu was first discovered, the molecular basis of the Dantu blood group antigen has been further resolved through whole genome sequencing (Leffler et al. 2017). It is now clear that Dantu consists of duplicate *GYPB-A* hybrid genes whose encoded protein contains the extracellular domain of glycophorin B and the transmembrane and intracellular domains of glycophorin A (Leffler et al. 2017). This molecular structure was further validated by fluorescent in situ hybridization using single-molecule DNA fibres (fibre-FISH) in lymphoblastoid cell lines (Algady et al. 2018). A recent study elucidated the inhibitory impact of Dantu on parasite invasion and, further, demonstrated that this protective effect was mediated by increased membrane tension (Kariuki et al. 2018). These functional studies have provided crucial insights into the biology of host-parasite interactions, and this biological knowledge is critical in developing novel intervention approaches for combating malaria.

## Conclusion

Malaria is the first, and arguably still remains the best, example of the impact that infectious diseases can have on the human genome. While numerous genes have now been identified that are strongly associated with the risk of different forms of malaria, it is those relating to the structure or function of RBCs for which the data are most compelling. This is entirely consistent with the fact that for all but a brief period during the incubation phase, the biological success of malaria parasites in humans is entirely dependent on their ability to invade, grow, and survive within RBCs. While some, including HbAS, the thalassaemias and G6PD deficiency, have been selected to extreme frequencies because of their malaria-protective effects, in many cases the mechanisms are either too poorly understood or too complex to suggest plausible approaches to the development of new treatments (Lelliott et al. 2015; Goheen, Campino, and Cerami 2017). Perhaps the most promising in this regard are polymorphisms in genes that are integral to the pathways by which parasites gain entry to red blood cells. Of particular current interest is the Dantu mutation in the glycophorin molecules that are important ligands in the parasite-invasion process. Remarkably however, the mechanism by which Dantu results in reduced invasion does not appear to be through a specific impact on receptor-ligand interactions but through a more non-specific mechanism whereby Dantu results in increased red cell tension. While further work is necessary, it is possible that drugs or small molecules could be developed with a view to inducing increased tension in non-Dantu subjects and thus providing therapeutic benefit in both treatment and prevention.
